# Editorial: Reflecting on Australia's health services response to the COVID-19 pandemic: what can be learned?

**DOI:** 10.3389/fpubh.2023.1199147

**Published:** 2023-05-02

**Authors:** Henryk Majewski, Sonja Pedell, Karen Willis

**Affiliations:** ^1^Independent Researcher, Balwyn North, VIC, Australia; ^2^Swinburne Living Lab, Centre for Design Innovation (CDI), School of Design and Architecture, Swinburne University of Technology, Hawthorn, VIC, Australia; ^3^Public Health, Institute for Health and Sport, Victoria University, Melbourne, VIC, Australia

**Keywords:** COVID-19, health service, pandemic, staff, remote (distant) education

COVID-19 swept the world at the beginning of 2020. The call for submissions for this special issue was at a point in time when Australia, by implementing lockdowns and external and internal border controls had minimized COVID in its communities. Once vaccines were rolled out (albeit somewhat delayed) high vaccination rates enabled the reopening of the Australian economy. While the overall COVID infection rate to date is broadly similar to other developed countries the case fatality rate is far lower than most European Countries and North America [but similar to Japan and South Korea–see (1)]. The call for papers was for learnings to be published on the effective health service responses which may be useful in future planning.

A key factor in Australia's COVID outcome was the public acceptance of vaccination at a time when there were mixed messages in social media about vaccine safety and the need for vaccination. The paper: “*Vaccine safety in Australia during the COVID-19 pandemic: lessons learned on the frontline*” describes the real-time safety surveillance system that was implemented and the need to create public confidence to support the vaccine rollout (Laemmle-Ruff et al.). Complementing this was a need to encourage vaccine use in marginalized communities approach as is described in “*Codesign and Community Outreach to Create COVID-19 Safe Communities: A Karen Community Case Study*” where a culturally and linguistically different (or CALD) migrant group was engaged in the overall community response (Davis et al.).

The second wave of the COVID-19 pandemic in Australia was more substantial than the first and caused significant workforce changes—these included rapid changes to protocols, shifts to telemedicine and redeployment of staff. A particular pressure point occurred in residential aged care services given the high risks of COVID fatality for older adults and the impact of lockdowns, staff shortages and lack of contact with family. As described in “*Adaptation among aged care and disability service providers in response to the COVID-19 pandemic: Lessons for the future*” considerable adaptation was required to provide COVID-safe services to vulnerable populations (Seivwright et al.). Organizations were required to be innovative to provide new or expanded services, to modify existing service delivery, and to adapt their organizational processes to meet the challenges of the pandemic.

Similarly, the study titled: “*Organizational responses to the COVID-19 pandemic in Victoria, Australia: a qualitative study across four healthcare settings”* found that health care workers valued organizational efforts to engage openly and honesty with staff. Improvement strategies included streamlined information processes, greater involvement of staff in decision-making, increased investment in staff wellbeing initiatives and sustainable approaches to strengthen the healthcare workforce (McGuinness et al.).

The themes of staff engagement and increased empowerment of staff, rather than a focus on management, in creating and delivering new service responses was also evident in teaching where universities had to maintain clinical education of future clinicians under very different circumstances. The experiences of doing so are described in **“***Burnout felt inevitable”: experiences of university staff in educating the nursing and allied health workforce during the first COVID-19 waves* (O'Brien et al.). In this paper, new service delivery methods as well as staff engagement and support were crucial in maintaining effective clinical education programs.

A shared theme in all contributions to the Special Issue is that staff in different settings had to work in an enhanced manner not only in delivery of services and activities but in a constantly changing environment. This included the stress of ensuring safe practice and risk mitigation for staff personally and for their clients. There was disrupted communications and disrupted support mechanisms for both staff and clients and the tensions created by rapid implementation of technology to fill service gaps. This happened simultaneously with the disruptive pressure that COVID brought to life away from work. In alignment with findings of Mehta et al. (2), the papers in this theme describe the stress and tension created by the COVID response in the healthcare workforce. In the initial call for papers, it was envisaged that this issue would collate a range of responses that can be shared and applied more widely. However, there has been little time for reflection and the health domain has moved on to its next emergency in many countries with staff shortages and strained health systems. Part of this is most likely a consequence of COVID. The workforce has had no time to recover from a herculean effort. This has resulted in many leaving their professions as well as burnout and disillusionment as seems to be the case in Australian State of Victoria which was most heavily hit by COVID (3). Compounding this, are the continuing health trends driven by demographics of an aging population particularly in developed countries and increased levels of chronic disease. COVID did not make this trend disappear and there is now an inevitable re-discovery of postponed or undiscovered health needs requiring treatment.

Despite the current situation, the papers in the theme give insights about innovative ways forward that should not be forgotten: The new ways of working and supporting staff, the better communication, the use of technology and the empowerment of staff to do things differently. The Editors hope the findings will be built on by other researchers. and maybe offer some solutions into current problems.

## Author contributions

All authors listed have made a substantial, direct, and intellectual contribution to the work and approved it for publication.
